# Hypomagnesemia, a Rare Cause of Reversible Ataxia

**DOI:** 10.5334/tohm.749

**Published:** 2023-05-04

**Authors:** Daniel López Domínguez, Juan Rodríguez Álvarez-Cienfuegos, Carla Herminia Vera Cáceres

**Affiliations:** 1 Movement Disorders Unit, Ataxia Unit, Neurology Department. Josep Tueta Hospital, Girona, Spain; 2 Stroke Unit, Neurology Department. Josep Tueta Hospital, Girona, Spain; 3 Neurology Resident, Neurology Department. Josep Tueta Hospital, Girona, Spain

**Keywords:** Ataxia, hypomagnesemia, neuroimaging

## Abstract

**Background:**

A 61-year-old male patient presented with cerebellar syndrome, which had progressively worsened for 10 days, followed by a tonic-clonic seizure.

**Phenomenology Shown:**

Blood analysis showed severe hypomagnesemia and a brain MRI showed T2 hyperintensity in the cerebellar hemispheres (Figure 1). Therefore, the final diagnosis was cerebellar syndrome and epileptic seizures secondary to severe hypomagnesemia.

**Educational Value:**

In cases of subacute onset of ataxia, the possibility of ataxia secondary to hypomagnesemia should be considered, as it can be diagnosed with a basic blood test and there are potentially life-threatening outcomes in the absence of treatment, with a reversible course following early supplementation.

Magnesium (Mg) is involved in multiple enzymatic processes [1], and an adequate concentration is essential for life. Mg deficiency is typically secondary to digestive/renal losses and has also been linked to proton pump inhibitors (PPI) [2]. Mg deficiency can produce various neurological manifestations, including movement disorders and seizures [1,3,4].

We report the case of a 61-year-old male with a history of heartburn (treated with PPI omeprazole) and systemic sclerosis with intestinal involvement, with secondary chronic diarrhea. He reported instability for 10 days, which progressively worsened until he was unable to walk. During his physical examination, we observed dysarthria, truncal ataxia and limb ataxia predominantly affecting the left side (18 points on the SARA scale). Afterwards, he presented with a tonic-clonic seizure, requiring hospital admission.

Given the rapidly progressing ataxia and epilepsy, we performed a brain MRI, which showed T2 hyperintensity in the cerebellar hemispheres (Figure 1), suggestive of vasogenic edema. Hence, we considered cerebrovascular etiology unlikely, given the progressive course and the absence of compatible findings in MRI. In light of the patient’s history of systemic sclerosis, we also considered autoimmune etiology, but discarded this option because of the absence of systemic symptoms and the normal results returned by a comprehensive autoimmunity assessment (including onconeural antibodies). A cereberospinal Fluid study showed no anomalies, all serologies were negative, and the patient never presented with fever; therefore, infectious etiology was ruled out.

**Figure 1:**
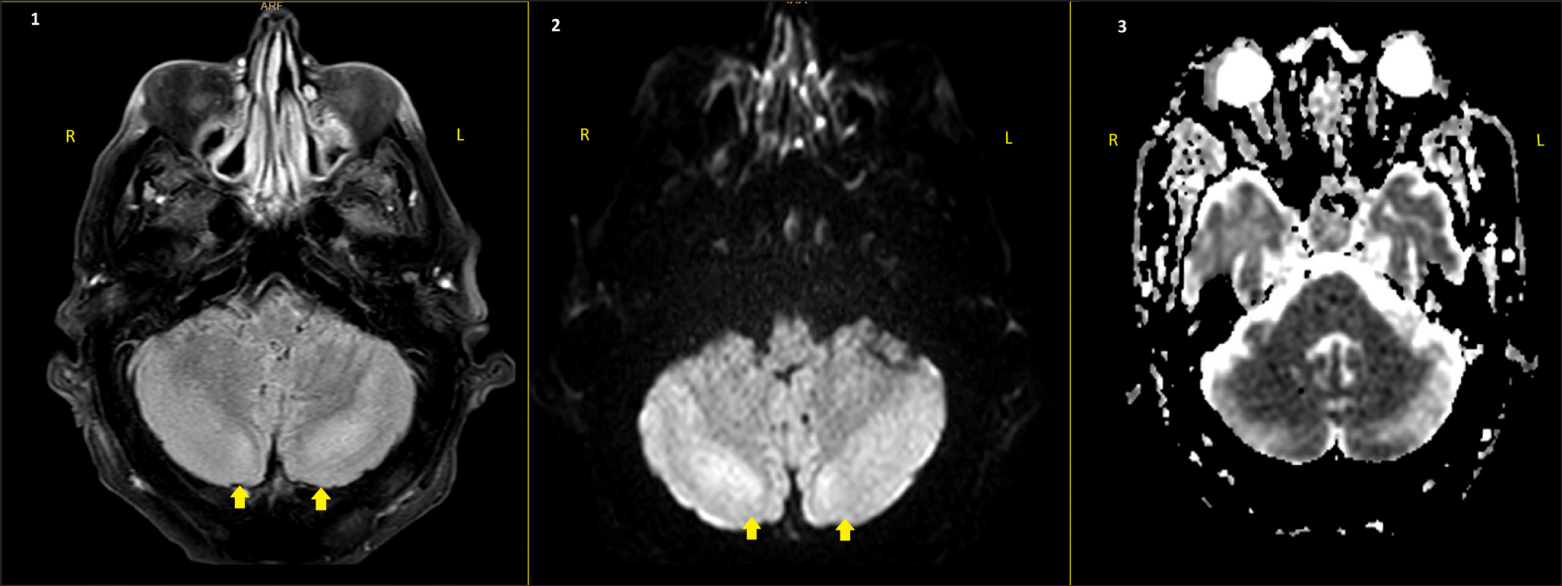
Brain MRI: **1.** Hyperintensity is observed on T2-FLAIR sequences in the bilateral cerebellar hemispheres (yellow arrows), with marked asymmetric (left > right) distribution. **2–3.** Hyperintensity is observed in DWI (2) and ADC (3) sequences, without clear restriction, which suggests of vasogenic edema at this level.

Finally, we considered toxic-metabolic etiology. A blood analysis revealed severe hypomagnesemia (0.31 mg/dL, normal levels 1.8–2.6 mg/dL), which was probably secondary to treatment with PPI and the chronic diarrhea. Therefore, we commenced supplementation, which corrected the patient’s Mg levels over the course of the following days, leading to progressive clinical improvement of the cerebellar syndrome, with only mild dysarthria persisting at hospital discharge (1 point on the SARA scale).

Therefore, the final diagnosis was cerebellar syndrome and epileptic seizures secondary to hypomagnesemia.

Cerebellar dysfunction secondary to hypomagnesemia has rarely been reported in the past [3]; and various authors have postulated that the dysfunction may be a distinct disease. This dysfunction typically presents with dysarthria and ataxia, with clinical improvement after correction of hypomagnesemia [1,3,4]. In neuroimaging, hyperintensities compatible with cerebellar edema [1] have been described in T2-weighted images. Various treatments have been tested, including corticotherapy and thiamine administration [1]; however, early Mg replacement is the only measure that has shown clinical and radiological improvements.

The pathophysiology is unknown. It has been proposed that endothelial dysfunction [1,2,3,5] could be the main factor contributing to cerebellar affection, with a mechanism similar to that proposed for Posterior Reversible Encephalopathy Syndrome (PRES) [5].

In the reported case, cerebellar ataxia was the initial manifestation of severe hypomagnesemia, preceding other major clinical complications by days. The neuroimaging findings, consistent with the clinical condition described, reaffirmed the suspicion. Early supplementation with Mg probably contributed to the good clinical response observed.

## Data Availability

All materials presented are accessible.

## Abbreviations

-PPI: proton pump inhibitor.

-CSF: Cerebrospinal fluid.

-MRI: Magnetic resonance.

-PRES: Posterior reversible encephalopathy syndrome.
